# Association of high-sensitivity C-reactive protein and uric acid with the metabolic syndrome components

**DOI:** 10.1186/s40064-016-1933-y

**Published:** 2016-03-03

**Authors:** Santosh Kumar Sah, Saroj Khatiwada, Sunil Pandey, Rajendra KC, Binod Kumar Lal Das, Nirmal Baral, Madhab Lamsal

**Affiliations:** Department of Biochemistry, Universal College of Medical Sciences, Bhairahawa, Nepal; Department of Biochemistry, Modern Technical College, Sanepa, Lalitpur, Nepal; Department of Biochemistry, B P Koirala Institute of Health Sciences, Dharan, Nepal

**Keywords:** High-sensitivity C-reactive protein, Metabolic syndrome, Nepal, Uric acid

## Abstract

Metabolic syndrome (MetS) has been found to be associated with inflammatory molecules. This study was conducted among 125 MetS patients at B P Koirala Institute of Health Sciences, Dharan, Nepal to find an association of high-sensitivity C-reactive protein (hs-CRP) and serum uric acid with MetS components. Anthropometric measurements, blood pressure, medical history and blood samples were taken. Estimation of hs-CRP, serum uric acid, blood glucose, triglyceride and high density lipoprotein (HDL) cholesterol was done. hs-CRP had positive correlation with blood glucose (r = 0.2, p = 0.026) and negative with HDL cholesterol (r = −0.361, p < 0.001). Serum uric acid had positive correlation with waist circumference (r = 0.178, p = 0.047). Patients with elevated hs-CRP and uric acid had higher waist circumference (p = 0.03), diastolic BP (p = 0.002) and lower HDL cholesterol (p = 0.004) than others. Elevated hs-CRP and high uric acid were individually associated with higher odds for low HDL cholesterol (7.992; 1.785–35.774, p = 0.002) and hyperglycemia (2.471; 1.111–5.495, p = 0.029) respectively. Combined rise of hs-CRP and uric acid was associated with severity of MetS (p < 0.001) and higher odds for hyperglycemia (8.036; 2.178–29.647, p = 0.001) as compared to individual rise of hs-CRP or uric acid. The present study demonstrates that hs-CRP and serum uric acid are associated with MetS components, and the combined rise of hs-CRP and uric acid is associated with the increase in severity of MetS.

## Background

Metabolic syndrome (MetS) is the clustering of several cardiovascular risk factors leading to increased risk of developing cardiovascular disease (CVD) and dying (Paoletti et al. 2006). MetS is a major and escalating public health and clinical challenge worldwide and is rising throughout the globe including developing countries like Nepal (Kaur 2014; O’Neill and O’Driscoll 2015; Sharma et al. 2011). Thus it is important to study the factors responsible for rapid rise of MetS and investigate markers that can predict risk for MetS early and the risk for future adverse events. Since, MetS is a state of chronic low grade inflammation as a consequence of complex interplay between genetic and environmental factors, inflammatory molecules seem to mediate the MetS components (Kaur 2014; Ma et al. 2012). Measurements of the circulating levels of the inflammatory molecules that accompany this syndrome might provide therapeutic approaches to modulate the inflammatory responses and thereby alter disease progression (Ma et al. 2012).

High-sensitivity C-reactive protein (hs-CRP) has been found to be associated with MetS. Hs-CRP is highly predictive of subsequent risk of cardiovascular events and diabetes mellitus in apparently healthy men and women (den Engelsen et al. 2012). Though, hs-CRP may serve as early marker for CVD and MetS, there is less evidence about relationship between hs-CRP level and the severity of MetS (as quantified by the increasing concurrence of individual MetS components; 3, 4 or 5), and there are evidences that addition of hs-CRP to the present definition of the MetS may help identify patients at high risk for future diabetes and CVD (Devaraj et al. 2009). Elevated serum uric acid level has been seen in MetS, and there appears to be link between MetS and serum uric acid (Serpa Neto et al. 2011). Hyperuricemia predicts the development of hypertension, obesity, and type 2 diabetes mellitus. Thus, uric acid may not only acts as surrogate marker of MetS, but also have a pathogenic role in the development of MetS, and therapies that lower uric acid may improve certain components of MetS (Billiet et al. 2014). Though an association between hyperuricemia and hs-CRP has been observed, the combined effect of hs-CRP and uric acid increase on MetS components is inadequately defined (Kawamoto et al. 2013). Thus it seems important to assess combined effects due to hs-CRP and uric acid rise on MetS parameters and to find if combined rise of hs-CRP and uric acid may have more severe effects on MetS than their individual rise.

Ethnicity has been suggested as a major factor in determining expression level of various metabolic markers and their association with CVD, type 2 diabetes and MetS (Abu-Farha et al. 2014). Studies investigating hs-CRP and uric acid in MetS are lacking in Nepalese population, and the clinical importance of measuring such markers has not been studied in Nepalese population. In the light of this gap in evidence from our settings and the undefined relationships between combined effects of h-CRP and uric acid rise on MetS components, we conducted a study among Nepalese MetS patients to explore the relationship between hs-CRP, serum uric acid and MetS components. The study also aims to evaluate the effect of the combined rise of hs-CRP and uric acid on MetS components.

## Methods

It was a cross-sectional study involving 125 MetS patients recruited at Department of Biochemistry of B P Koirala Institute of Health Sciences, Nepal. The aim was to find an association between hs-CRP, serum uric acid and MetS components, and to determine whether combined increase of hs-CRP and serum uric acid is associated with MetS components. The study patients were recruited from the B P Koirala Institute of Health Sciences conveniently using the study inclusion criteria. Following informed consent, blood pressure (BP) were measured using standard methods and 5 ml of fasting blood samples were collected from the patients whose waist circumference were ≥90 cm in men and ≥80 cm in women. Only patients fulfilling International Diabetes Federation (IDF) criteria for MetS were enrolled in the study. According to this criteria; for a person to be defined as having the MetS they must have; central obesity (defined as waist circumference with ethnicity specific values, for South Asians: waist circumference ≥90 Cm for males and ≥80 Cm for females), plus any two of the following four factors: triglycerides ≥150 mg/dL or on treatment for lowering this lipid, high density lipoprotein (HDL) cholesterol <40 mg/dL in men or <50 mg/dL in women or on treatment for HDL cholesterol, systolic BP ≥130 mm Hg or diastolic BP ≥85 mm Hg or on treatment for hypertension, fasting blood glucose ≥100 mg/dL or on treatment for type 2 diabetes (Kaur 2014). Raised BP or hypertension, raised glucose or hyperglycemia, hypertriglyceridemia and low HDL was considered if the subject’s systolic BP ≥130 mm Hg or diastolic BP ≥85 mm Hg, fasting blood glucose ≥100 mg/dL, triglycerides ≥150 mg/dL and HDL cholesterol <40 mg/dL in men or <50 mg/dL in women respectively (Kaur 2014). Exclusion criteria of the participants include: presence of acute infections and chronic inflammatory diseases, gout patients. After defining MetS, each patient demographic characteristics (age, sex), height, weight, medical history of diabetes mellitus and hypertension were noted. The study protocol was approved by the institute review board of B P Koirala Institute of Health Sciences.

Serums from overnight fasting blood samples were stored at −20 °C until analysis. Blood glucose was estimated using enzymatic method. Serum triglyceride was measured by enzymatic method (kits from AGAPPE diagnostics). High density lipoprotein (HDL) cholesterol was estimated by homogeneous, direct method using kit from Gesan by Biolyzer 100, and hs-CRP was measured by enzyme immunoassay using kit from diagnostics biochem canada (DBC) and the reading was taken using LabLife ER 2007 plate reader. Uric acid was measured by uricase peroxidase method. We dichotomized hs-CRP (hs-CRP <1 mg/L as non-elevated hs-CRP and low risk group, and hs-CRP ≥1 mg/L as elevated hs-CRP and high risk group) and serum uric acid (serum uric acid <6 mg/dL as normal uric acid group and serum uric acid ≥6 mg/dL as hyperuricemia group) and analyzed MetS components in each group. We used hs-CRP <1 or ≥1 mg/L and serum uric acid <6 or ≥6 mg/dL as a cutoff. Usually, hs-CRP ≥ 1 mg/L, indicates intermediate or high cardiovascular risk, whereas, hs-CRP <1 mg/L is considered a low cardiovascular risk (den Engelsen et al. 2012). We took uric acid ≥6 mg/dL as high uric acid/hyperuricemia since exact cutoff of serum uric acid for hyperuricemia in Nepalese population has not been established, and uric acid level even at upper normal range could bring risk for having MetS components, and some define hyperuricemia as uric acid >6 mg/dL (women) and >6.8 mg/dL (men) (Billiet et al. 2014; Serpa Neto et al. 2011). Similarly, we compared MetS components among 4 groups made by considering both hs-CRP and serum uric acid; group one with hs-CRP < 1 mg/L and normal uric acid, group two with hs-CRP < 1 mg/L and hyperuricemia, group three with hs-CRP ≥ 1 mg/L and normal uric acid and group four with hs-CRP ≥ 1 mg/L and hyperuricemia.

The data were analyzed using SPSS version 20. The continuous variables were expressed as mean ± SD for variables showing normal distribution, and as median with interquartile rage for variables showing non-normal distribution. Categorical variables are expressed as number (percentage). Correlations between hs-CRP and MetS components were calculated using Spearman rank correlation analysis, and between serum uric acid level and MetS components using Pearson’s correlation analysis at 95 % confidence interval. Correlation between hs-CRP and uric acid level was measured using Spearman rank correlation analysis at 95 % confidence interval. Independent t test, one way ANOVA, Man Whitney test and Kruskal–Wallis test were applied at 95 % confidence interval to test for the significance. The odds ratio with 95 % CI was estimated to find risk for having hypertension, hyperglycemia, hypertriglyceridemia and low HDL in patients with elevated hs-CRP, hyperuricemia or both as compared to those with low hs-CRP, uric acid or both respectively. A *p* value less than 0.05 was considered as statistically significant at 95 % confidence interval.

## Results

The mean age of the study population was 47.5 ± 11.1 years (age range 22–73 years). Males and females were 44 % (n = 55) and 56 % (n = 70) respectively. History of diabetes mellitus and hypertension was present in 55.2 % (n = 69) and 76 % (n = 95) respectively. Mean height, weight and BMI of study population were 158.5 ± 8.2 Cm, 70.1 ± 9.6 kg and 27.9 ± 3.4 kg/m^2^ respectively. The mean level of MetS components; waist circumference, systolic BP, diastolic BP, fasting blood glucose, triglyceride and HDL cholesterol were 103.8 ± 7.4 Cm, 128.8 ± 13.3 mmHg, 84.1 ± 10.6 mmHg, 128.9 ± 48.9 mg/dL, 208.2 ± 80.7 mg/dL and 50.3 ± 13.2 mg/dL respectively. The median hs-CRP and mean serum uric acid level was 2.5 mg/L (1 mg/L; 5.1 mg/L) and 6.6 ± 1.8 mg/dL respectively. No significant difference in MetS components was observed among male and female patients except for waist circumference (male 106 ± 5.4 Cm and female 102.1 ± 8.4 Cm; p = 0.003) and HDL cholesterol (male 47.2 ± 11.9 mg/dL and female 52.7 ± 13.6 mg/dL, p = 0.018). MetS components according to level of hs-CRP and uric acid is shown in Table 1. Significant difference in MetS components; systolic BP (p = 0.043), diastolic BP (p = 0.005) and HDL cholesterol (p < 0.001) was observed among patient with hs-CRP < 1 mg/L and hs-CRP ≥ 1 mg/L. However, only significant difference (p = 0.009) in MetS component; waist circumference was observed among patients with normal uric acid and hyperuricemic patients. MetS components in the 4 groups made by considering level of hs-CRP and uric acid is shown in Table 2. There was significant rise in waist circumference and diastolic BP and significant decrease in HDL cholesterol across the groups.

**Table 1:** MetS components according to hs-CRP and uric acid level

MetS components	Non-elevated hs-CRP, N = 25	Elevated hs-CRP, N = 100	P value	Uric acid <6 mg/dL, N = 55	Uric acid ≥6 mg/dL, N = 70	P value
Waist circumference (Cm)	101.8 ± 6.3	104.3 ± 7.6	0.14	101.9 ± 6.2	105.3 ± 8	0.009
Systolic BP (mmHg)	124 ± 13.2	130 ± 13.1	0.043	127.5 ± 14.5	129.9 ± 12.3	0.318
Diastolic BP (mmHg)	78.8 ± 11.2	85.5 ± 10.1	0.005	82.5 ± 10.6	85.4 ± 10.5	0.121
Fasting blood glucose (mg/dL)	114.4 ± 41.7	132.5 ± 50.1	0.097	121.1 ± 50.3	135.1 ± 47.2	0.113
Triglyceride (mg/dL)	188.7 ± 55.7	213.1 ± 85.3	0.087	210.4 ± 89.9	206.6 ± 73.2	0.795
HDL cholesterol (mg/dL)	58.6 ± 12	48.2 ± 12.6	<0.001	50.9 ± 12.9	49.8 ± 13.4	0.648

The data is expressed as mean ± SD

*MetS* metabolic syndrome, *hs-CRP* high sensitivity C reactive protein, *HDL* high density lipoprotein

**Table 2:** MetS components according to combined hs-CRP and uric acid level

MetS components	Non-elevated hs-CRP and uric acid <6 mg/dL, n = 14	Non-elevated hs-CRP and uric acid ≥6 mg/dL, N = 11	Elevated hs-CRP and uric acid <6 mg/dL, n = 41	Elevated hs-CRP and uric acid ≥6 mg/dL, n = 59	P value
Waist circumference (Cm)	101.4 ± 5.9	102.4 ± 7	102 ± 6.3	105.9 ± 8.1	0.03
Systolic BP (mmHg)	123.9 ± 14.6	124.1 ± 12	128.7 ± 14.4	130.9 ± 12.2	0.19
Diastolic BP (mmHg)	81.4 ± 12.2	75.5 ± 9.3	82.8 ± 10.2	87.3 ± 9.7	0.002
Fasting blood glucose (mg/dL)	98.6 ± 44.5	134.5 ± 28.4	128.8 ± 50.4	135.2 ± 50.1	0.089
Triglyceride (mg/dL)	198.9 ± 53.5	175.6 ± 58.2	214.3 ± 99.6	212.3 ± 74.7	0.505
HDL cholesterol (mg/dL)	58.6 ± 14.7	58.7 ± 8	48.2 ± 11.2	48.1 ± 13.6	0.004

The data is expressed as mean ± SD

*MetS* metabolic syndrome, *hs-CRP* high sensitivity C reactive protein, *HDL* high density lipoprotein

The correlation of hs-CRP with MetS components; waist circumference, systolic BP, diastolic BP, fasting blood glucose, triglyceride and HDL cholesterol were 0.15 (p = 0.095), −0.008 (p = 0.931), 0.072 (p = 0.426), 0.2 (p = 0.026), 0.124 (p = 0.168) and −0.361 (p < 0.001) respectively. The correlation of serum uric acid with waist circumference, systolic BP, diastolic BP, fasting blood glucose, triglyceride and HDL cholesterol were 0.178 (p = 0.047), 0.099 (p = 0.271), 0.121 (p = 0.181), 0.144 (p = 0.109), 0.095 (p = 0.294) and −0.034 (p = 0.704). Significant correlation (r = 0.19, p = 0.034) was observed between hs-CRP and serum uric acid level.

Among the study population 3, 4 and 5 components of MetS was present in 66, 37 and 22 patients respectively. All 5 components were very common in patients (19 patients out of 22) with elevated hs-CRP and hyperuricemia than compared to 4 (15 patients out of 37) and 3 (25 patients out of 66) components. Combined rise of hs-CRP and uric acid was associated with severity of MetS (p < 0.001). Median hs-CRP and mean uric acid level in patients with 3, 4 and 5 components were 1.1 mg/L (1; 4) and 6.3 ± 1.7 mg/dL, 3.2 mg/L (1.6; 5.6) and 6.5 ± 1.7 mg/dL, and 4.4 mg/L (2.1; 8.1) and 7.8 ± 1.8 mg/dL respectively. Hs-CRP (p < 0.001) and uric acid level (p = 0.005) were significantly different among those with 3, 4 and 5 components.

Among the study patients, hypertension, hyperglycemia, hypertriglyceridemia and low HDL cholesterol were observed in 76 % (n = 95), 72 % (n = 90), 80 % (n = 100) and 34.4 % (n = 43) patients respectively. The prevalence rate of raised BP, hyperglycemia and low HDL cholesterol was higher in group with hs-CRP ≥ 1 mg/L than hs-CRP < 1 mg/L, and in hyperuricemia group than normal uric acid group. However, hypertriglyceridemia rate remained similar across the group. Odds ratio of having hypertension, hyperglycemia, hypertriglyceridemia and low HDL cholesterol in patients with elevated hs-CRP as compared to those with non-elevated hs-CRP, and in patients with hyperuricemia as compared to those with normal uric acid level is shown in Table 3. Significant odds of having low HDL cholesterol were observed in patients with elevated hs-CRP as compared to those with non-elevated hs-CRP. Hyperuricemic patients have significant odds of having hyperglycemia as compared to patients with normal uric acid level. The odds ratio of having hypertension, hyperglycemia, hypertriglyceridemia and low HDL cholesterol in patients with non-elevated hs-CRP and hyperuricemia, elevated hs-CRP and normal uric acid, and elevated hs-CRP and hyperuricemia as compared to the patients with non-elevated hs-CRP and normal uric acid is shown in Table 3. Patients with elevated hs-CRP and normal uric acid, and with elevated hs-CRP and hyperuricemia had significant odds of having hyperglycemia as compared to those with low hs-CRP and normal uric acid.

**Table 3:** Odds ratio for MetS components in relation to hs-CRP and uric acid level

MetS components	Non-elevated hs-CRP, N = 25	Elevated hs-CRP, N = 100	Uric acid <6 mg/dL, N = 55	Uric acid ≥6 mg/dL, N = 70	Non-elevated hs-CRP and uric acid <6 mg/dL, n = 14	Non-elevated hs-CRP and uric acid ≥6 mg/dL, N = 11	Elevated hs-CRP and uric acid <6 mg/dL, n = 41	Elevated hs-CRP and uric acid ≥6 mg/dL, n = 59
Raised BP	17 (68 %)	78 (78 %)	39 (70.9 %)	56 (80 %)	10 (71.4 %)	7 (63.6 %)	29 (70.7 %)	49 (83 %)
OR (95 % CI)	1 (reference)	1.668 (0.636–4.377, p = 0.304)	1 (reference)	1.641 (0.719–3.747, p = 0.293)	1 (reference)	0.7 (0.129–3.791, p = 1.0)	0.967 (0.253–3.694, p = 1.0)	1.96 (0.511–7.517, p = 0.449)
Raised glucose	15 (60 %)	75 (75 %)	34 (61.8 %)	56 (80 %)	4 (28.5 %)	11 (100 %)	30 (73.1 %)	45 (76.2 %)
OR (95 % CI)	1 (reference)	2.0 (0.798–5.015, p = 0.144)	1 (reference)	2.471 (1.111–5.495, p = 0.029)	1 (reference)	–	6.818 (1.768–26.294, p = 0.005)	8.036 (2.178–29.647, p = 0.001)
Hypertriglyceridemia	20 (80 %)	80 (80 %)	44 (80 %)	56 (80 %)	13 (92.8 %)	7 (63.6 %)	31 (75.6 %)	49 (83 %)
OR (95 % CI)	1 (reference)	1.0 (0.334–2.991, p = 1)	1 (reference)	1.0 (0.414–2.418, p = 1.0)	1 (reference)	0.135 (0.013–1.449, p = 0.133)	0.238 (0.028–2.058, p = 0.255)	0.377 (0.044–3.219, p = 0.679)
Low HDL	2 (8 %)	41 (41 %)	18 (32.7 %)	25 (35.7 %)	2 (14.2 %)	–	16 (39 %)	25 (42.3 %)
OR (95 % CI)	1 (reference)	7.992 (1.785–35.774, p = 0.002)	1 (reference)	1.142 (0.542–2.408, p = 0.85)	1 (reference)	–	3.84 (0.758–19.465, p = 0.11)	4.412 (0.906–21.494, p = 0.067)

The data is expressed as number (percentage). Odds ratio was calculated at 95 % confidence interval

*MetS* metabolic syndrome, *hs-CRP* high sensitivity C reactive protein, *HDL* high density lipoprotein

## Discussion

South Asians are more prone to develop MetS because of their high percentage of body fat, abdominal obesity, and insulin resistance (Mahalle et al. 2014). Hypertension, MetS and diabetes mellitus has been rising in Nepalese population. Accompanying with the rise of chronic diseases like diabetes mellitus, MetS and chronic kidney diseases, there is increase in endocrine disorders like thyroid dysfunction, dyslipidemia and CVD in Nepal (Khatiwada et al. 2015a, b, 2016; Sharma et al. 2011). The present study was conducted among MetS patients in eastern Nepal. All of the study patients were obese (waist circumference 103.8 ± 7.4 Cm). The mean level of MetS components was similar to our previous study among MetS patients in eastern Nepal (Khatiwada et al. 2016). Among the gender, females had significantly lower waist circumference and HDL cholesterol than male patients. Epidemiological research has shown that there is gender disparity among these components of MetS. In a study in Pakistan, similar finding among gender was reported (Jahan et al. 2007).

The median hs-CRP level (2.5 mg/L) in our study population was in risk zone and similar to the values seen in earlier studies. In a study in India, median levels of hs-CRP were considerably higher in individuals with MetS (Mahajan et al. 2012). In study by Engelsen et al., median hs-CRP levels were significantly higher in individuals with central obesity with the MetS compared to individuals with central obesity without the metabolic syndrome (2.2 versus 1.7 mg/L, p < 0.001) (den Engelsen et al. 2012). In the present study, we observed that median hs-CRP level increases significantly with increasing number of MetS components. Thus, patients with all 5 MetS components have higher hs-CRP than those with 4 and 3 components. This finding is similar to that reported by Nishida et al., who reported that individuals with an increased number of MetS components have higher CRP levels, and by Yan et al., who demonstrated increase in hs-CRP among Uyghurs and Kazakhs as the clustering of MetS components increased (Yan et al. 2015). We observed significant positive correlation between hs-CRP and fasting blood glucose, and significant negative correlation of hs-CRP with HDL cholesterol. A number of studies have observed an association of hs-CRP with various MetS components (Abu-Farha et al. 2014; DeBoer et al. 2011; den Engelsen et al. 2012). In study in non–Hispanic black adolescents in US, with the exception of diastolic BP and fasting glucose, hs-CRP was significantly correlated with each of the components of MetS, however in a study among Arab population significant correlation of hs-CRP was observed with all components of MetS (Abu-Farha et al. 2014; DeBoer et al. 2011).

In our study cohort, we observed significantly higher BP (systolic and diastolic) and lower HDL cholesterol, and increasing prevalence of hypertension, hyperglycemia and low HDL cholesterol with the rise in hs-CRP. Similarly, patients with elevated hs-CRP showed higher risk for hypertension, hyperglycemia and low HDL cholesterol as compared to patients with non-elevated hs-CRP. Similar trends were observed in the study by Yan et al., where prevalence of MetS and its components increased across the quartiles of hs-CRP level, and risk for MetS and its components were higher for fourth quartile as compared to first quartile of hs-CRP (Yan et al. 2015). Another study by Kawamoto et al., also observed that prevalence rate of MetS and its components increased significantly in relation to the higher tertiles of hs-CRP, and odds ratios of hs-CRP were significantly high for obesity, hypertriglyceridemia, low HDL cholesterolemia and impaired fasting glucose (Kawamoto et al. 2013). The mechanisms by which hs-CRP reflect the risk for MetS are not completely understood. However, systemic inflammation is closely involved in the pathogenesis of MetS, and thus, elevated hs-CRP may also reflect inflammation, which impairs insulin signaling in the liver, muscle, and adipose tissues, and influence insulin resistance, lipid and glucose metabolism (Kawamoto et al. 2013). Further investigations are required to find the molecular role of CRP in the pathogenesis of MetS.

The mean serum uric acid (6.9 ± 1.6 mg/dL for male and 6.4 ± 1.9 mg/dL for female) in our study population was higher than the normal range for males and females. Previous studies have also reported higher serum uric acid level in MetS patients than compared to non-MetS patients (Nejatinamini et al. 2015; Numata et al. 2008). In the current study, we found significantly higher waist circumference in patients with hyperuricemia as compared to patients with normal uric acid level and a significant positive correlation of serum uric acid with waist circumference. Study by Nejatinamini et al. (2015) and Li et al. (2011) have found strong correlation between serum uric acid and MetS components.

Our findings show that serum uric acid level increases with increase in severity of MetS, and prevalence of hypertension, hyperglycemia and low HDL cholesterol increases with the rise in serum uric acid. Also, hyperuricemic patients have higher odds of having hypertension, hyperglycemia and low HDL cholesterol as compared to patients with normal serum uric acid level. A number of previous studies have reported such findings (Lin et al. 2006; Liu et al. 2014). In the study by Lin et al., serum uric acid level was elevated significantly as the number of metabolic components increased (Lin et al. 2006). In a study in China, the prevalence of MetS increased with rise in serum uric acid and MetS component number showed an increasing trend across serum uric acid quartile in both sexes (p for trend < 0.01). Similarly, participants with hyperuricemia or higher serum uric acid level were at significantly elevated odd ratios for MetS and most of its five individual components (Liu et al. 2014). While it has been suggested that uric acid may simply be a consequence of the increased uric acid absorption in the proximal tubule secondary to hyperinsulinemia, there is growing data that uric acid may predict the development of MetS, obesity and diabetes. Although a strong relationship between hyperuricemia and metabolic syndrome has been established through animal and epidemiological studies, the potential pathophysiological mechanisms by which uric acid contributes to this disease state is not clear (Soltani et al. 2013).

In the present study, we reported that combined rise of hs-CRP and uric acid is associated with significant increase in level of MetS components (waist circumference and diastolic BP) and significant decrease in HDL cholesterol than individual rise of either hs-CRP or uric acid, and the prevalence and risk for hypertension, hyperglycemia and low HDL cholesterol is higher in patients with combined rise of hs-CRP and uric acid than in patients with either raised hs-CRP or uric acid. Also, the severity of MetS tends to be higher in patients with raised hs-CRP and raised uric acid than in patients with raised hs-CRP or uric acid, and the combined rise of hs-CRP and serum uric acid occurs more frequently in patients with higher number of MetS components. These findings are in accord to a previous study by Kawamoto et al., who found that the odds ratio of an increased tertile of uric acid and hs-CRP was a significant and independent determinant for MetS and insulin resistance. In addition to their direct associations, they also observed a synergistic effect between uric acid and hs-CRP (Kawamoto et al. 2013). Since, we observed strong association of hs-CRP with serum uric acid level in the present study, which is similar to findings of Kawamoto et al., where uric acid levels significantly increased with hs-CRP (r = 0.285, p < 0.001), it may be possible that hs-CRP and uric acid act synergistically to increase severity of MetS and risk for further complications like diabetes mellitus and CVD (Kawamoto et al. 2013). Hyperuricemia has been found associated with elevated CRP and other inflammatory markers and there might be link between uric acid and hs-CRP (Krishnan 2014). In a study in China, hs-CRP was correlated with uric acid, and there was significant rise in prevalence of hyperuricemia with rise of hs-CRP (Sun et al. 2015).

To our knowledge, we report the association of hs-CRP and serum uric acid with MetS components for the first time in Nepalese population. Both hs-CRP and uric acid can act as an indicator of inflammation, which may negatively affect the MetS components thereby increasing CVD risk. Our previous study in eastern Nepal reported higher preponderance of CVD risk factors in subclinical hypothyroidism patients who had high hs-CRP levels than control having low hs-CRP (Kc et al. 2015). Our findings suggest that measurement of hs-CRP and uric acid may be helpful to assess severity of MetS. Because of the elevated hs-CRP in MetS patients and its ability to predict future adverse outcomes such as diabetes mellitus and CVD in MetS, adding hs-CRP as a clinical criterion for MetS may be advantageous (Devaraj et al. 2009).

The present study has some limitations, which should be considered. First, the study sample size was small, thus there might be effects on generalization of our findings. Since, this was a cross-sectional study one cannot overtly draw conclusion about the casual relationships between hs-CRP, uric acid and MetS components. Also, we did not studied hs-CRP and uric acid in subjects without any MetS components.

## Conclusion

Our study finds an association of hs-CRP and serum uric acid with MetS components, though serum uric acid seems to be associated with fewer MetS components than hs-CRP. Rise of hs-CRP, uric acid, or both tends to associated with increased risk for hypertension, hyperglycemia and low HDL cholesterol. Similarly, combined rise of hs-CRP and uric acid is associated with the increase in severity of MetS. Thus, hs-CRP and uric acid may be used to assess severity of MetS.

## Abbreviations

BMI: body mass index
BP: blood pressure
CVD: cardiovascular disease
HDL: high density lipoprotein
hs-CRP: high sensitivity C reactive protein
IDF: International Diabetes Federation
LDL: low density lipoprotein
MetS: metabolic syndrome
